# Key Role of Transfer Layer in Load Dependence of Friction on Hydrogenated Diamond-Like Carbon Films in Humid Air and Vacuum

**DOI:** 10.3390/ma12091550

**Published:** 2019-05-12

**Authors:** Yunhai Liu, Lei Chen, Bin Zhang, Zhongyue Cao, Pengfei Shi, Yong Peng, Ningning Zhou, Junyan Zhang, Linmao Qian

**Affiliations:** 1 Tribology Research Institute, State Key Laboratory of Traction Power, Southwest Jiaotong University, Chengdu 610031, China; liuyun@my.swjtu.edu.cn (Y.L.); pengfei_s@my.swjtu.edu.cn (P.S.); pengyong@my.swjtu.edu.cn (Y.P.); linmao@swjtu.edu.cn (L.Q.); 2 State Key Laboratory of Solid Lubrication, Lanzhou Institute of Chemical Physics, Chinese Academy of Sciences, Lanzhou 730000, China; caozhongyue10@mails.ucas.ac.cn (Z.C.); zhangjunyan@licp.cas.cn (J.Z.); 3 Beijing Key Laboratory of Long-life Technology of Precise Rotation and Transmission Mechanisms, Beijing Institute of Control Engineering, Beijing 100094, China; zhzhining@163.com

**Keywords:** hydrogenated DLC film, superlubricity, environment dependence, transfer layer, normal load

## Abstract

The friction of hydrogenated diamond-like carbon (H-DLC) films was evaluated under the controlled environments of humid air and vacuum by varying the applied load. In humid air, there is a threshold applied load below which no obvious friction drop occurs and above which the friction decreases to a relatively low level following the running-in process. By contrast, superlubricity can be realized at low applied loads but easily fails at high applied loads under vacuum conditions. Further analysis indicates that the graphitization of the sliding H-DLC surface has a negligible contribution to the sharp drop of friction during the running-in process under both humid air and vacuum conditions. The low friction in humid air and the superlow friction in vacuum are mainly attributed to the formation and stability of the transfer layer on the counterface, which depend on the load and surrounding environment. These results can help us understand the low-friction mechanism of H-DLC film and define optimized working conditions in practical applications, in which the transfer layer can be maintained for a long time under low applied load conditions in vacuum, whereas a high load can benefit the formation of the transfer layer in humid air.

## 1. Introduction

Nowadays, in order to reduce the huge space launch costs, recoverable carrier rockets, space shuttles and other recoverable spacecraft have been major areas of focus for industries and research groups from all over the world. However, the reliability and service life of the key components of the recoverable spacecraft are greatly affected due to the need to repeatedly traverse the atmosphere and vacuum environments. Therefore, it is necessary to develop an applicable coating to protect the key parts applied under these two conditions. Diamond-like carbon (DLC) films are regarded as one of the most promising space lubricating materials due to their excellent physical, chemical and tribological properties [1,2,3,4,5]. Nevertheless, the high sensitivity of the friction behaviors of DLC films to the working environment seriously restricts their practical applications, especially in the aerospace industries [6,7,8,9]. Thus, it is essential to probe the effects of room air and vacuum, which respectively simulate the earth and space environments, on the friction behaviors of DLC films. 

Although the comparison of friction on DLC films in air and vacuum has attracted extensive interest during recent decades, the environmental dependence of friction behaviors described in the present literature is heterogeneous, and the corresponding mechanisms are far from understood. Initially, the friction behaviors in air and vacuum were found to strongly depend on the hydrogen content in DLC film. For instance, in Andersson et al. [10], compared to that in humid air, the friction coefficient of hydrogen-free DLC film was higher in vacuum owing to strong covalent bond interactions, whereas a highly hydrogenated DLC film presented a lower friction coefficient in vacuum owing to hydrogen passivation. Vanhulsel and Donnet et al. [11,12] also observed a lower friction coefficient of H-DLC film in vacuum than in humid air. A similar impact caused by water vapor was demonstrated by Liu et al. [13], who found that an increased in relative humidity (RH) decreased the friction coefficient of hydrogen-free DLC film but increased the friction coefficient of H-DLC film. 

However, the opposite environmental dependence of the friction behavior was reported in other studies. Both Yang et al. [14] and Konca et al. [15] observed significantly higher friction coefficients of H-DLC films in vacuum than in humid air. They indicated that friction tests in humid air might benefit the smoothing of track surfaces and the formation of the transfer layer, resulting in a low friction force. Grill et al. [16] deduced that inadequate hydrogen passivation in vacuum induced a high friction coefficient of H-DLC film with low hydrogen content. Later, Xia et al. [17] demonstrated that the material of the counterface played a significant role in the environmental dependence of friction on the H-DLC surface. Compared to that in dry air, the friction of H-DLC film in vacuum was lower when sliding against bearing steel owing to the oxidization of the steel counterparts, but was higher when sliding against Si_3_N_4_ balls owing to the dehydrogenation and graphitizing of the H-DLC film.

In this paper, the friction behaviors of H-DLC films are compared in humid air and vacuum with the variation of a normal load. The results show that the friction behaviors of the H-DLC film strongly depend on not only the surrounding environment but also the contact pressure (normal load). Insights gained from this study are helpful for enriching the understanding of the low-friction mechanism of H-DLC films and promoting the practical applications of these films, especially in the space industries.

## 2. Experimental Material and Methods

### 2.1. Preparation of H-DLC Film

H-DLC film was prepared on a silicon surface by the plasma-enhanced chemical-vapor deposition method. The hydrogen content of the pristine H-DLC film was estimated to less than ~20% based on the Raman spectrum simulated by an empirical formula [18]. In the deposition procedures, the silicon wafers were ultrasonically cleaned with acetone and ethanol for 30 min following drying in a dry chamber at first; then the cleaned wafers were placed into the vacuum chamber (10^−4^ Pa) and etched by Ar+ ions at a pressure of 5 Pa for 30 min, not only to remove the native oxides and surface contamination but also to improve the bonding strength between the substrate and the deposited material. After that, to optimize the adhesion strength between the film and substrate, a ~55 nm thick nitrogen doped H-DLC film as a buffer layer was first grown on the silicon wafers in working atmosphere of a mixture of methane and nitrogen. Finally, the H-DLC film was deposited as lubricating layer in the mixed gases of methane and hydrogen [19]. The film thickness was measured as around 870 nm based on observation of the cross section using a scanning electron microscope. 

### 2.2. Test Methods

The friction behaviors of H-DLC films against Al_2_O_3_ balls (radius = ~1.5 mm) were compared in humid air with a relative humidity (RH) of 25 ± 3% and vacuum with a pressure of less than 10^−3^ Pa at room temperature using a rotational ball-on-disk tribometer (CSM tribometer, CSM Instruments, Peseux, Switzerland). Before each test, all samples were cleaned ultrasonically in ethanol (C_2_H_5_OH) for about 5 min to eliminate the surface contamination. The sliding velocity used in the tests was kept at 200 mm/s. The used normal loads of 1, 3, and 5 N corresponded to initial mean Hertzian contact pressures of 884, 1275, and 1511 MPa, respectively. Each friction measurement was repeated three times under the same conditions to minimize data scattering. After the sliding tests, the worn surfaces of the H-DLC films were measured by a white light interferometer (ADE Phase Shift, ClassOne Equipment, Decatur, GA, USA), and the topographies inside the worn regions were scanned by an atomic force microscope (AFM, E-sweep, Hitachi, Tokyo, Japan). The surface features of the Al_2_O_3_ balls were characterized by 3D profilometry (MFT-3000, Rtec, San Jose, CA, USA) and an optical microscope. The chemical structures of the worn surfaces of the H-DLC films and the transfer layers covered on the Al_2_O_3_ ball surfaces were analyzed with a Raman spectroscope (Jobin-Yvon HR-800, Jobin Yvon Company, Lille, France).

## 3. Results 

### 3.1. Characterization of H-DLC Film

**Figure 1:**
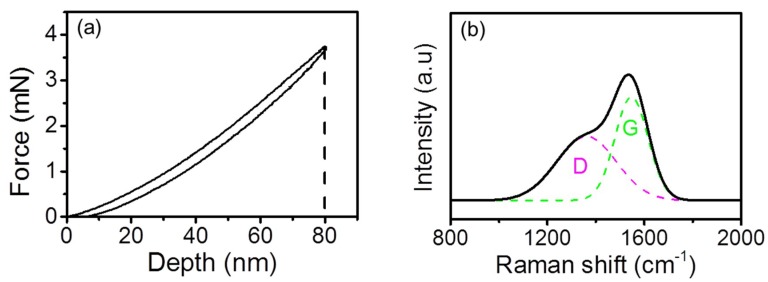
(**a**) Indentation force-depth curve of H-DLC film with maximum depth of 80 nm. (**b**) Raman spectrum of pristine H-DLC film. Two peaks involving D peak at ~1335 cm^−1^ and G peak at ~1540 cm^−1^ were deduced.

The Figure 1a shows the indentation force-depth curve of the H-DLC film as measured using a nanoindenter (Hysitron, TI 750, Minneapolis, MN, USA). Since the maximum indentation depth is 10 times less than the film thickness (~870 nm), the intrinsic hardness and elastic modulus of the H-DLC film can be characterized as ~11.6 and ~117.3 GPa, respectively. The bonding structure of the H-DLC film was measured by a Raman spectroscope (Jobin-Yvon HR-800, Jobin Yvon Company, Lille, France). As shown in Figure 1b, the appearance of two peaks involving the D peak at ~1335 cm^−1^ and the G peak at ~1540 cm^−1^ indicates that the sample is a typical DLC film. The intensity ratio (I_D_/I_G_) of the H-DLC film is estimated as ~0.62. Using an atomic force microscope (AFM, E-sweep, Hitachi, Tokyo, Japan), the root-mean-square (RMS) roughness of the H-DLC film surface was measured as ~0.18 ± 0.04 nm (averaged at nine different locations) in a scanning area of 1 × 1 μm. The intrinsic properties of the H-DLC film are summarized in Table 1.

### 3.2. Friction Behaviors of H-DLC Films in Humid Air and Vacuum

**Figure 2:**
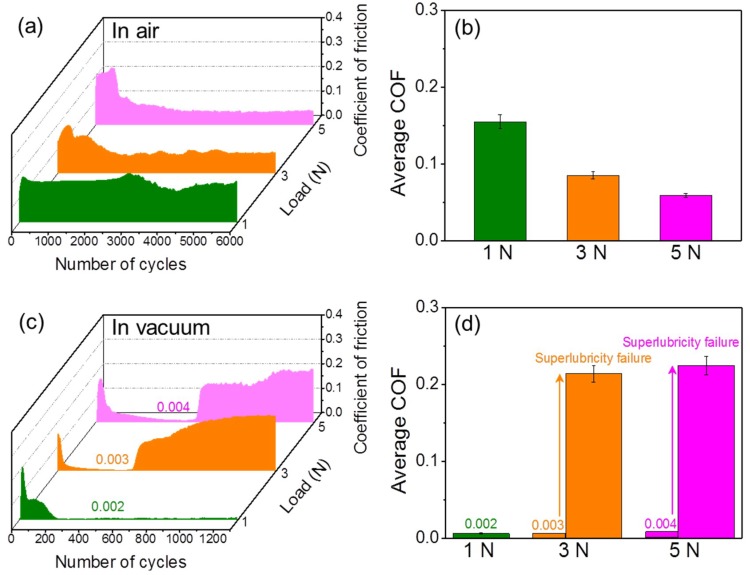
Friction behaviors of H-DLC films against Al_2_O_3_ balls at different normal loads in (**a**,**b**) humid air and (**c**,**d**) vacuum. (**a**) and (**b**) respectively show sliding cycle dependence of friction coefficient and average friction coefficients in steady region as function of normal load in humid air. (**c**) and (**d**) respectively show friction coefficient curves and average friction coefficients in vacuum.

The friction behaviors of the H-DLC films against Al_2_O_3_ balls in humid air and vacuum were compared at various applied loads. As shown in Figure 2, the variation of the friction coefficient not only relates to the surrounding environment but also strongly depends on the mechanical interaction applied to the contact interface. In humid air, the friction coefficients at only high applied loads (≥3 N) go through a running-in period and then level off at relatively low steady-state values (Figure 2a). No obvious drop of friction occurs at 1 N. The average friction coefficients in the steady region can be estimated as ~0.06–~0.15 depending on the applied loads (Figure 2b). By contrast, the friction coefficients decrease dramatically after a very short running-in process (~200 cycles), and then reach an extremely low friction level (<0.004) in vacuum (Figure 2c), achieving superlubricity [20]. The minimum friction coefficient of the H-DLC film against an Al_2_O_3_ ball is found to decrease to ~0.002 at a load of 1 N (contact pressure = ~884 MPa). However, high contact pressure can facilitate the failure of the superlubricity state. After around 600–800 sliding cycles, the friction coefficients at loads of 3 and 5 N increase quickly to ~0.22 and then maintain this value in the subsequent sliding process, indicating the failure of superlubricity (Figure 2c,d). It is intriguing that with an increase in the normal load, the stable friction coefficients after the running-in process decrease gradually from ~0.15 to ~0.06 in humid air, but increase (before or after superlubricity failure) from ~0.002 to ~0.004 (before superlubricity failure) or ~0.22 (after superlubricity failure) in vacuum. This opposite variation implies that the lubrication mechanisms of H-DLC films against Al_2_O_3_ balls in humid air and vacuum should be diverse.

### 3.3. Wear Behaviors of H-DLC Films in Humid Air and Vacuum

**Figure 3:**
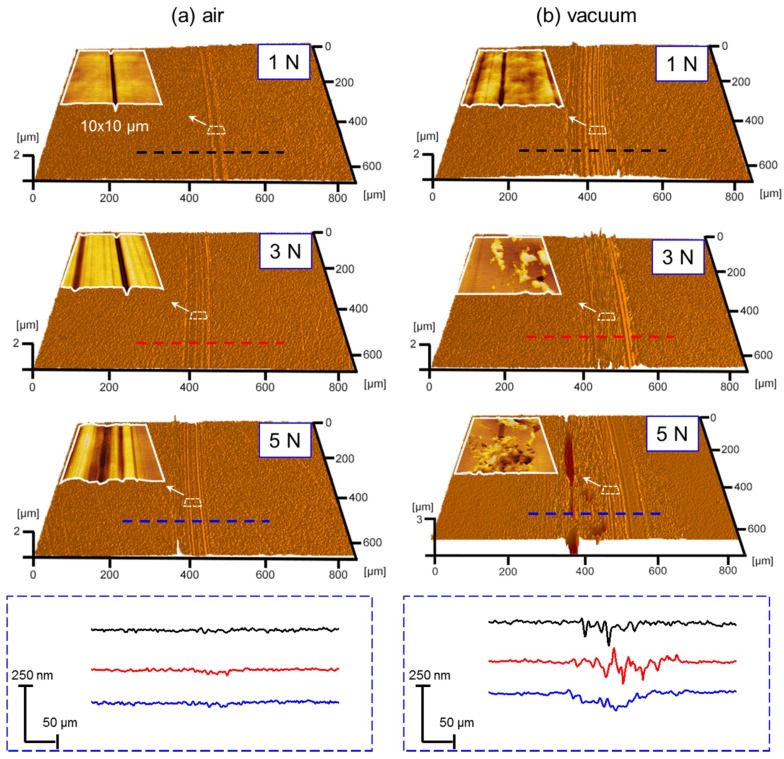
Three-dimensional images and corresponding cross-section profiles of wear tracks on H-DLC films formed at different normal loads in (**a**) humid air and (**b**) vacuum. Upper-left inset in each topographical picture shows AFM image inside wear track with scan area of 10 × 10 μm.

Not only the friction behaviors but also the surface wear of H-DLC films depend on the surrounding environment. Figure 3 displays the topographies and corresponding cross-section profiles of the worn scars formed after 6000 sliding cycles under various conditions. In humid air, the H-DLC film presents excellent wear resistance. Only slight surface wear occurs, even at a normal load of 5 N (Figure 3a). By contrast, although the sliding system reaches the superlubricity state in vacuum, serious surface damage occurred on the H-DLC surface (Figure 3b) under the superlubricity condition (1 N) or the superlubricity failure conditions (3 and 5 N). Additional details in the worn regions were characterized using AFM with a sharp Si_3_N_4_ tip (radius = ~15 nm). The representative AFM image in the worn region with a scan area of 10 × 10 μm^2^ is shown as the upper-left inset in each topographical picture. Scratches along the sliding directions can be observed under all given conditions. Compared to the cases in humid air, the worn surfaces present significantly different features in vacuum, especially under high-load conditions (i.e., 3 and 5 N in Figure 3b). Pits are formed along the scratches, and considerable wear debris is produced under these two conditions. Based on the AFM images, the RMS roughness values of the worn surfaces in a 10 × 10 μm^2^ scanning region can be respectively estimated as ~2.8–~12.5 nm in humid air and ~18.6–~269.3 nm in vacuum as the normal load ranges from 1 N to 5 N. The much rougher worn surface in vacuum eliminates the possibility that the superlubricity originates from the formation of matching contact interface.

## 4. Discussion

Similar to the results reported in previous studies, the surrounding environment can significantly change the friction characteristics of H-DLC film depending on the contact pressure. Here, we found that the superlubricity of the H-DLC film in vacuum was vulnerable to failure at relatively high loads, but low friction in humid air could be maintained in the long term under the same load conditions. Based on the present friction mechanisms of DLC films, which involve DLC substrate graphitization [21,22], transfer layer formation [23,24,25], and surface passivation [26,27,28], the evolution of the sliding interface (the substrate surface and the counterface) should determine the friction behaviors on the H-DLC surface in different environments. 

### 4.1. Structure Evolution of H-DLC Film after Sliding in Humid Air and Vacuum

**Figure 4:**
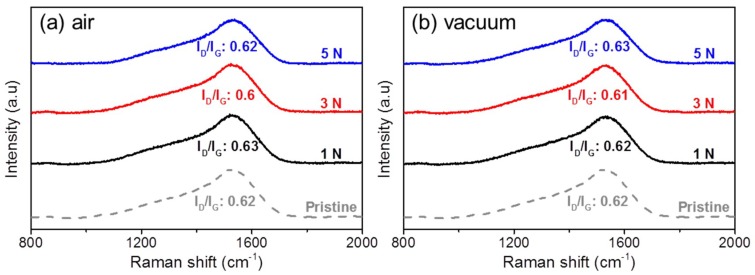
Raman spectra of worn surfaces on H-DLC films formed at various applied loads in (**a**) humid air and (**b**) vacuum.

Raman analysis was used to detect possible changes in the worn surface response to the different friction behaviors of H-DLC films against Al_2_O_3_ balls in humid air and vacuum. Figure 4 shows the Raman spectra measured in the worn regions of H-DLC films (Figure 3) formed at various load conditions under these two environments. The structure of H-DLC film depends on the ratio of sp^3^ and sp^2^, which can be defined by the ratio of *D* and *G* bonds (*I*_D_/*I*_G_). Physically, the D peak is due to the breathing modes of sp^2^ bonded atoms in the carbon rings [22,29], while the G peak is due to the in-plane bond stretching of all pairs of sp^2^ bonded atoms in both rings and chains [18,30]. Therefore, an increase in *I*_D_/*I*_G_ corresponds to graphitization of the DLC film [18]. It is interesting that the *I*_D_/*I*_G_ values on all worn surfaces of H-DLC films stay at 0.62 ± 0.02, regardless of the environment and load conditions (Figure 4). This is very close to the value measured on a pristine H-DLC surface (Figure 1). This indicates that a change in material structure in the worn regions of H-DLC surfaces is below the detecting limitation of Raman tests. Previous studies reported that DLC film can present low friction owing to graphitization of the sliding substrate surface [21,22]. However, the undetectable variation of *I*_D_/*I*_G_ shown in Figure 4 implies that the graphitization of the sliding H-DLC surface is negligible in this study. Thus, its contribution to the sharp drop in friction during the running-in process can be ruled out. Similar phenomena were reported in other studies [31,32,33]. 

### 4.2. Formation of Transfer Layer on The Counterface and Environment Dependence

**Figure 5:**
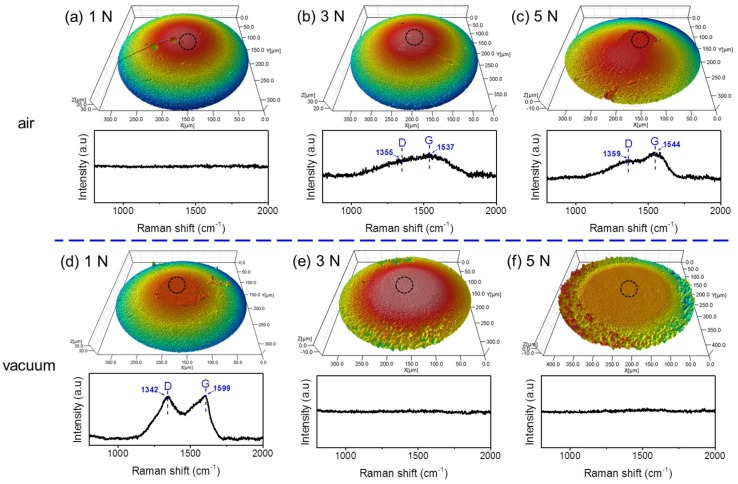
Three-dimensional images of (top) Al_2_O_3_ balls and (bottom) Raman spectra in contact regions on ball surfaces after sliding 6000 cycles in humid air (**a–c**) and vacuum (**d–f**). Loads were 1, 3, and 5 N, respectively.

Since there was no remarkable structural change on the worn surface of H-DLC film during the sliding process, the difference between the friction behaviors in humid air and vacuum should be strongly related to changes in the counterface. To reveal these changes, the topographies of the Al_2_O_3_ balls as well as the Raman spectra in the contact regions on the ball surfaces were compared under different conditions (Figure 5). In humid air, a transfer layer was found to form on the Al_2_O_3_ ball surfaces after sliding 6000 cycles at normal loads of 3 and 5 N. However, this did not occur at 1 N (upper 3D images in Figure 5a). These topographical changes at high load conditions are consistent with the results of Raman spectra measured in the contact regions, where the *D* and *G* bands only appeared at 3 and 5 N (bottom Raman spectra in Figure 5b,c). A similar result was reported in another study, where the transfer layer was difficult to generate on the counterface at low contact pressures (<1.0 GPa) in air [34]. This should be the reason for the lack of an obvious friction drop during the sliding process at a load of 1 N in humid air. Once the transfer layer formed on Al_2_O_3_ ball surfaces (Figure 5b,c), the friction forces decreased sharply during the initial ~2000 cycles (the conditions of 3 and 5 N in Figure 2a). 

In vacuum, the topography of the Al_2_O_3_ ball surface as well as the Raman spectrum in the contact region after testing at 1 N indicate that a transfer layer is formed on the counterface at this low normal load (Figure 5d). Compared to humid air, the vacuum environment seems to facilitate the material transferring from H-DLC film to the counterface at a low normal load. Furthermore, the value of *I*_D_/*I*_G_ increases to ~0.9 (Figure 5d), which is much higher than that of the pristine H-DLC surface (0.62, Figure 1b). This result indicates that high graphitization in the transfer layer occurs during the sliding process in vacuum, which may result in a sharp drop in friction to reach a superlubricity state (Figure 2c). However, no signals of Raman spectra were observed at 3 and 5 N, although superlow frictions were achieved during the running-in process. The three-dimensional images show material removal instead of a transfer layer on the Al_2_O_3_ ball surface under these two conditions (Figure 5e,f). One reasonable explanation is that a highly graphitized transfer layer is subjected to removal under high mechanical interaction, thus inducing a further increase in the friction coefficient owing to superlubricity failure (Figure 2d).

**Figure 6:**
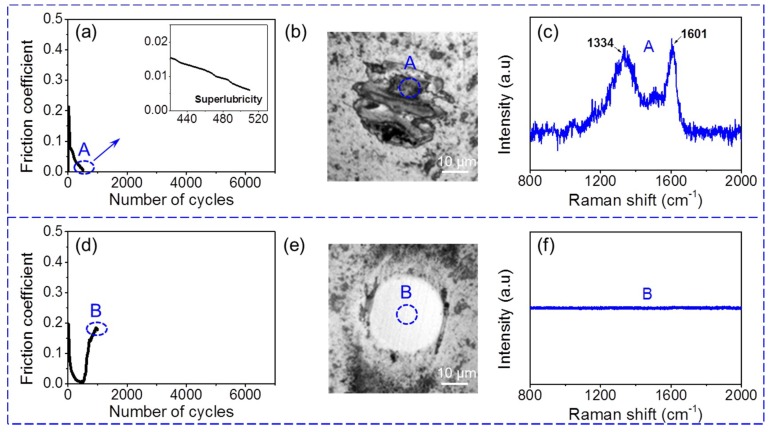
Characterizations of Al_2_O_3_ ball surface after sliding ~510 cycles and ~1000 cycles at 5 N in vacuum. (**a**) Friction coefficient decreases to superlubricity state at ~510 sliding cycles. (**b**,**c**) respectively show topography of used Al_2_O_3_ ball and Raman spectrum measured in contact region during superlubricity stage. (**d**) Friction coefficient increases with superlubricity failure. (**e**,**f**) show topography and Raman spectrum of Al_2_O_3_ ball surface in this stage.

In order to prove this hypothesis, the counterface in the stage of superlubricity achieved at 5 N (Figure 6a–c) was characterized by optical topography and Raman analysis to compare with the cases after superlubricity failed (Figure 6d–f). When the friction coefficient decreases to ~0.005 at ~510 sliding cycles (Figure 6a), a notable transfer layer can be observed in the contact region on the Al_2_O_3_ ball surface (Figure 6b). The Raman spectrum measured in the contact region shows a high graphitization transfer layer (Figure 6c). By contrast, significant material removal occurs on the Al_2_O_3_ ball (Figure 6e), and the Raman signal disappears completely (Figure 6f), as the superlubricity fails at ~1000 sliding cycles. These results confirm that the removal of the transfer layer should be the main contributor to a high friction coefficient at a high normal load following a superlubricity failure in vacuum.

The friction behaviors of H-DLC films in Figure 2 and the counterface features in Figure 5 indicate that the transfer layer plays a significant role in the reduction of the friction coefficient in humid air and vacuum. This is very different from the results given by Arnell [35], who found that water molecules from humid air might adsorb at the dangling carbon bonds on the edge-faces of the sp^2^ clusters in the DLC coating, resulting in lower friction coefficient at higher RH conditions. Moreover, Akaishi et al. [36] also found that a double-layer structure of water molecules forms on the graphitic surface, which may result in a low friction. Here, the superlubricity state can be achieved only under vacuum condition. The Raman spectra in Figure 5b,c show that the G peak upshifts from ~1540 cm^−1^ in humid air to ~1600 cm^−1^ in vacuum. A previous study identified that an upshift of the G peak over ~1595 cm^−1^ corresponded to the formation of curved graphene ribbons and onion carbon nanoparticles in the transfer layer, which can result in superlubricity in the sliding interface [34]. Therefore, the difference environment in friction behaviors between humid air and vacuum may be attributed to the diverse structure of the formed transfer layer.

### 4.3. Stability of Low-Friction State on H-DLC Surface in Humid Air and Vacuum 

In summary, H-DLC film presents a superlow friction coefficient (<0.01) in vacuum, but the lifetime of superlubricity is very limited at high-load conditions (i.e., 3 and 5 N in Figure 2c) owing to the stress-induced removal of the transfer layer. After superlubricity failure, the friction coefficient increases significantly, and considerable wear occurs on the Al_2_O_3_ counterface (Figure 5e,f). By contrast, a transfer layer cannot be formed at a low load (i.e., 1 N) in humid air. However, the formed transfer layer presents good resistance under high-load conditions, resulting in stable lubrication in the H-DLC/Al_2_O_3_ interface (Figure 2a and Figure 4b,c). This indicates that the stability of the low-friction state on the H-DLC surface strongly depends on not only the surrounding environment but also the contact pressure. In other words, H-DLC film can provide excellent lubricating properties at high contact pressure in humid air, whereas durable superlubricity is realized at a low contact pressure in vacuum.

**Figure 7:**
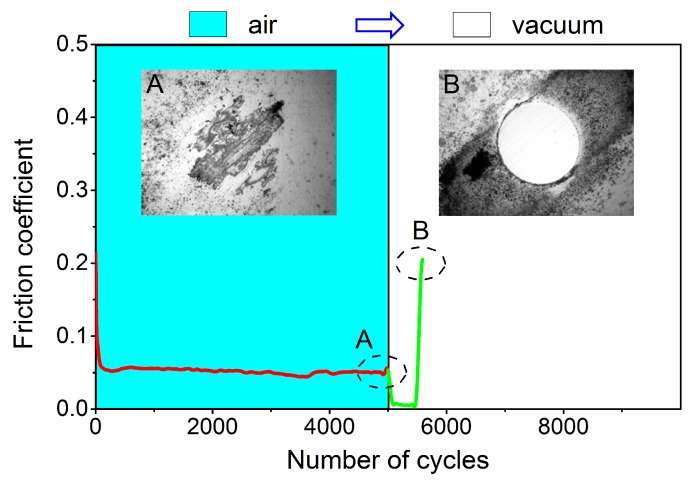
Friction behaviors of H-DLC film slid against Al_2_O_3_ ball in humid air following sliding tests in vacuum (10^−3^ Pa) after ~5000 cycles. Upper insets compare topographical images of Al_2_O_3_ ball after sliding tests in humid air (**A**) and vacuum (**B**). Sliding velocity and normal load were maintained at 200 mm/s and 10 N during entire experiment, respectively.

Another experiment was conducted to further confirm the stability of lubricity in different environments. As shown in Figure 7, a new Al_2_O_3_ ball was first modified by repeated rubbing cycles on the H-DLC surface in humid air. During the initial running-in process, the friction coefficient decreased to a stable value of ~0.45. At the same time, an obvious transfer layer could be observed on the counterface (left inset). After that, friction tests between the modified ball and the worn H-DLC surface were performed in situ under vacuum condition as a comparison. The friction coefficient further decreased to ~0.02 but increased sharply after ~500 sliding cycles. Similar to the cases shown in Figure 2c and Figure 5e,f, the lubricity failed rapidly at high loads in vacuum owing to the removal of the transfer layer following counterface wear (right inset in Figure 7).

Comparing the friction behaviors in humid air and vacuum, the durability of transfer layers covered on the counterface may relate to their different structures as well as diverse surface passivation. On one hand, the curved graphene ribbons and onion carbon nanoparticles formed in vacuum may decrease the adhesive strength of the transfer layer on the counterface, resulting in the removal of the transfer layer at relatively high normal loads. On the other hand, the H-DLC film surface is saturated by the reservoir of hydrogen atoms of H-DLC films [26,27,37]. Then, the hydrogen passivation in vacuum has to change as gas passivation in humid air [6,28,35,38]. The adsorbed water molecules from air can saturate the friction-induced carbon *σ*-bonds at the sliding interface, which may enhance the adhesive interaction of the transfer layer itself and with the counterface [38,39], thus ensuring a stable transfer layer on the counterface [40,41,42]. 

**Figure 8:**
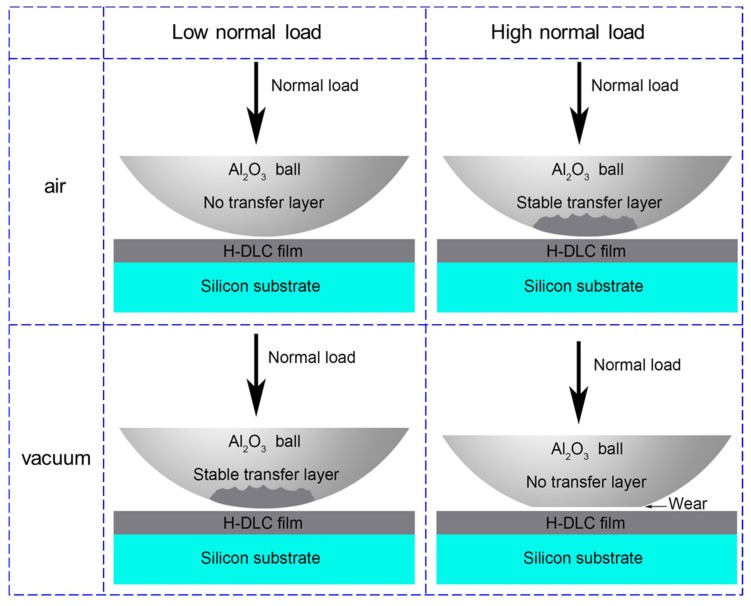
Schematics showing formation and stability of transfer layer on counterface after sliding H-DLC films at different load conditions in humid air and vacuum.

In summary, Figure 8 schematically shows the effects of an environmental atmosphere and normal load on the formation and stability of the transfer layer on the sliding counterface during the running-in process. At a low normal load, no obvious material was transferred from the H-DLC film to the contact surface of the Al_2_O_3_ ball in humid air (upper left picture in Figure 8), whereas the formed transfer layer resulted in superlow friction under vacuum condition (bottom left picture). At high normal loads, a transfer layer could form and lubricate the sliding interface with good durability in humid air (upper right picture). By contrast, the formed transfer layer during the initial running-in process was removed from the counterface owing to the high contact pressure in vacuum, resulting in the wear of the Al_2_O_3_ ball and superlubricity failure (bottom right picture).

## 5. Conclusions

The effect of the environmental atmosphere on the friction behaviors of H-DLC film was investigated in the present study. The main conclusions are as follows:

(1) The load dependence of friction behaviors on H-DLC film strongly depends on the surrounding environment. The running-in stage only occurs at high normal load conditions following a relatively low-friction coefficient in humid air. By contrast, the friction can decrease to a superlubricity state at low load conditions but can easily fail at high loads in vacuum.
(2) The contribution of H-DLC substrate surface graphitization to a sharp drop in friction was ruled out in all cases. In humid air, the transfer layer on the counterface formed at relatively high loads plays a key role in the sharp drop of the friction coefficient. In vacuum, the transfer layer can be formed at low load conditions, resulting in a superlow friction coefficient. However, the formed transfer layer cannot be maintained for a long time at relatively high loads.
(3) The lifetime of the low-friction state after the running-in process is determined by the formation and stability of the transfer layer on the counterface under both humid air and vacuum conditions. The friction coefficient decreases to a relatively low level at a high enough load in humid air. Conversely, the superlubricity realized in vacuum can only be maintained for a long time at a low load.

## Figures and Tables

**Table 1:** Intrinsic properties of H-DLC film.

Sample	Thickness	RMS Roughness	Hardness	Elastic Modulus	I_D_/I_G_
H-DLC film	~870 nm	~0.18 ± 0.04 nm	~11.6 GPa	~117.3 GPa	~0.62
